# Supplementary material for: Suppression of B-Cell Activation by Human Cord Blood-Derived Stem Cells (CB-SCs) through the Galectin-9-Dependent Mechanism
Source: Int J Mol Sci. 2024 Feb 2;25(3):1830. doi: 10.3390/ijms25031830 (PMC10855911; doi:10.3390/ijms25031830)
Supplement: Supplementary file 1 [file ijms-25-01830-s001.zip › ijms-2841306-supplementary.pdf]

Supplementary data

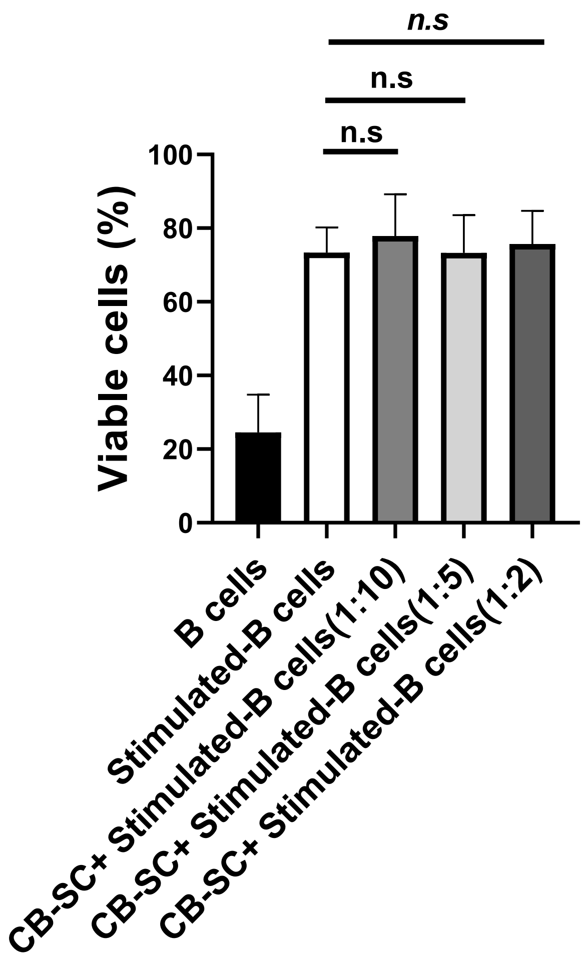

Figure S1: Effects of B cell death after treatment with different ratioes of CB-SC. No increased in dead cell rate could be detected in activation cocktails treated group with or without CB-SC.

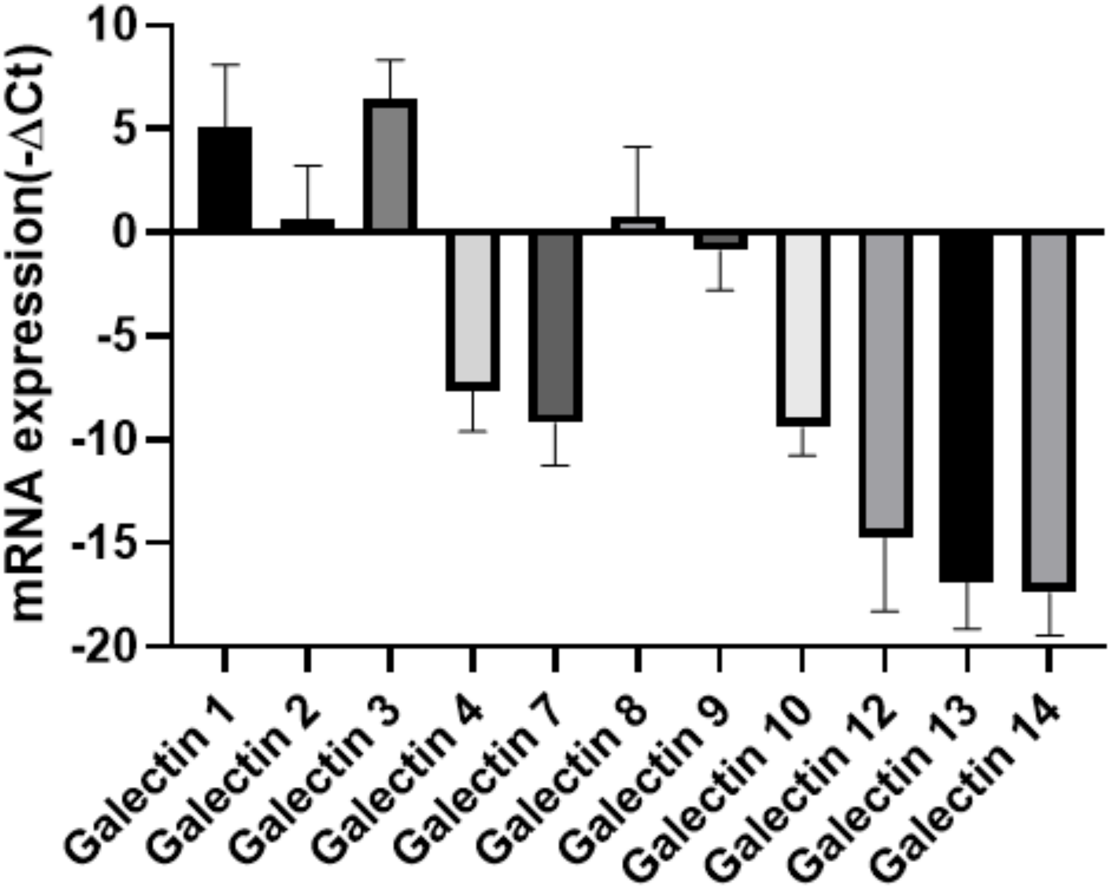

Figure S2: RT-PCR analysis of galectin expressions in CB-SC (N = 4). β actin served as control.

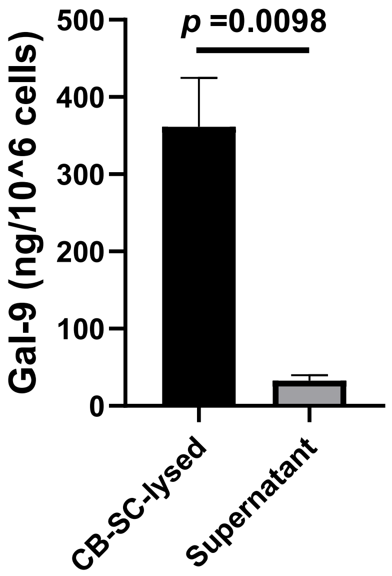

Figure S3: Galectin-9 expression in CB-SC and CB-SC culture supernatant. CB-SC released galectin-9 were less than 10% percentage of CB-SC contained galectin-9 after 3 days culture.

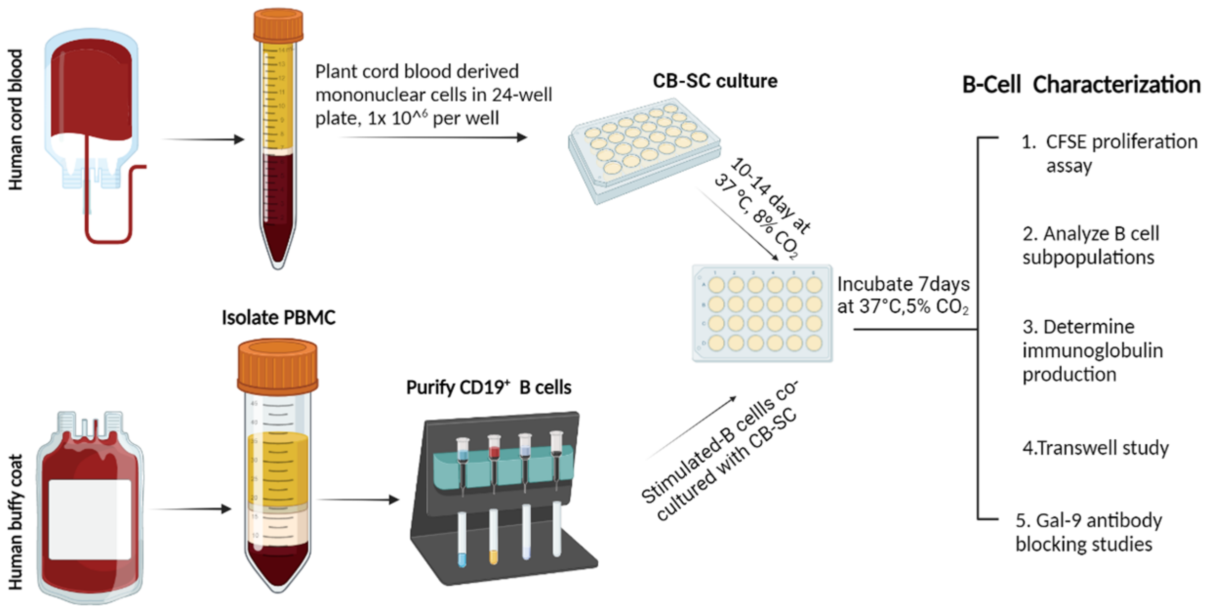

Figure S4. Outline of the whole protocol to determine the immune modulation of CB-SC on B cells.

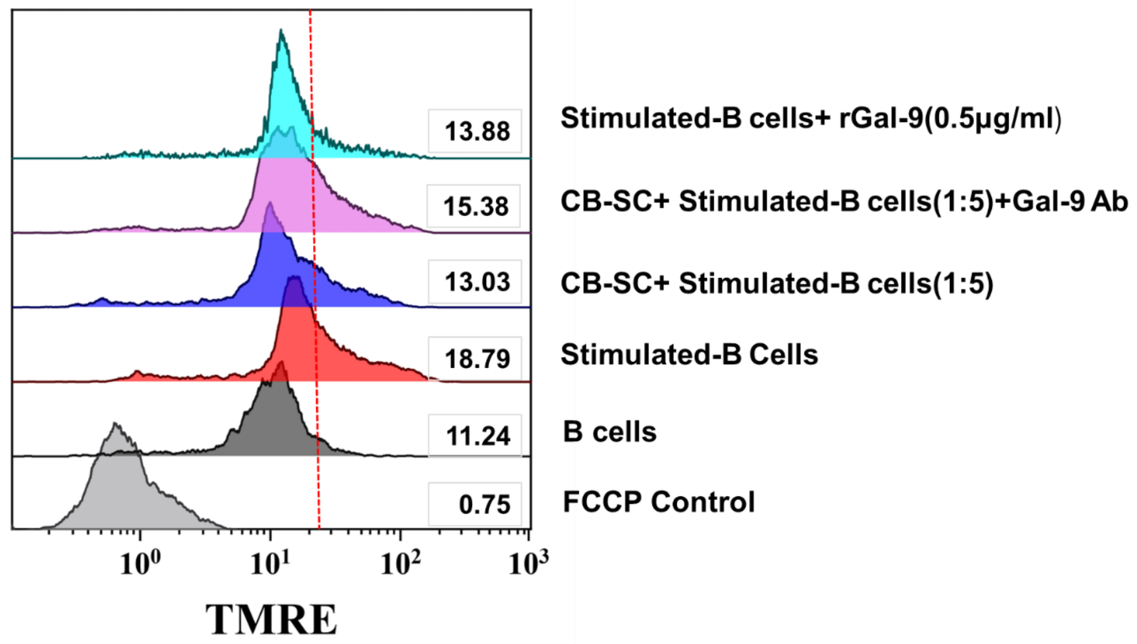

Figure S5. Flow cytometry analysis of TMRE staining in B cells. The data showed the increase in the mitochondrial membrane potential (Δ ψm) in the stimulated B cells (red) relative to that of the unstimulated B cells control (dark grey). A reduction in the mitochondrial membrane potential was noticed with the stimulated B cells after the treatment with CB-SC (dark blue), and such a reduction was almost completely recovered after the blocking treatment with Gal-9 mAb (pink).

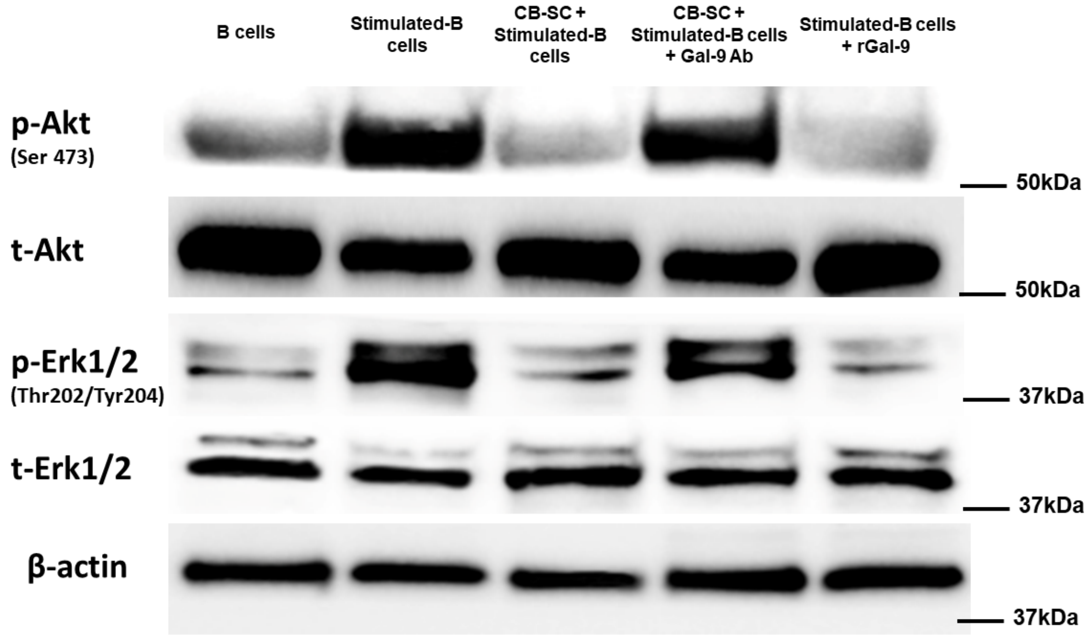

Figure S6. Western blotting showed the reduced expression of phosphorylated AKT and ERK1/2 in stimulated B cells after the treatment with CB-SC or rGal-9, but upregulated after blocking with Gal-9 mAb. β-actin served as control.

Table S1. List of Reagents:

No	Name of Reagents	Vendor/Manufacturer	Catalogue
1	CD19 MicroBeads, human	Miltenyi Biotec	130-050-301
2	Ficoll-Paque® PLUS Medium (1.076 g/ml Density; 1.078 g/ml Density Max.)	VWR	95038-168
3	X-VIVO™ 15 Serum-free Hematopoietic Cell Medium	Lonza	BP04-744Q
4	AffiniPure F(ab')$_2$ Fragment Goat Anti-Human IgM, Fc5μ fragment specific	Jackson Immunoresearch Laboratory Inc	109-006-129
5	Recombinant Human CD40L	BioLegend	591706
6	Recombinant Human IL-2	BioLegend	589104
7	Recombinant Human IL-10	BioLegend	571004
8	Recombinant Human IL-21	BioLegend	571202
9	CellTrace™ CFSE Cell Proliferation Kit, for flow cytometry	ThermoFisher Scientific	C34554
10	TheraPEAKTM ACK Lysing Buffer	Lonza	BP10-548E
11	RNeasy Mini Kit	Qiagen	74106
12	iScript™ gDNA Clear cDNA Synthesis Kit	BioRad	1725034
13	LEGENDplex™ Human Immunoglobulin Isotyping Panel (6-plex) with V-bottom Plate	Biolegend	740640
14	Purified anti-human Galectin-9 Antibody	Biolegend	348902
15	Fluo-4, AM, cell permeant	ThermoFisher Scientific	F14201
16	Rhod-2, AM, cell permeant	ThermoFisher Scientific	R1244
17	TMRE-Mitochondrial Membrane Potential Assay Kit	Abcam	ab113852
18	Phospho-Akt (Ser473) (1:2000 dilution)	Cell Signaling Technology	9271S
19	Akt Antibody (1:2000 dilution)	Cell Signaling Technology	9272S
20	p44/42 MAPK (Erk1/2) Antibody (1:2000 dilution)	Cell Signaling Technology	9102S
21	Phospho-p44/42 MAPK (Erk1/2) (Thr202/Tyr204) Antibody (1:2000 dilution)	Cell Signaling Technology	9101S
22	β-Actin (13E5) Rabbit mAb (HRP Conjugate) (1:2000 dilution)	Cell Signaling Technology	5125
23	PhosSTOP	Millipore Sigma	4906845001
24	Goat anti-Rabbit IgG (H+L) Secondary Antibody, HRP (1:10000 dilution)	ThermoFisher Scientific	31460

25	CD45-PC7 (10 µL/sample at 1:10 dilution)	Beckman Coulter	IM3548U
26	OCT3/4 Monoclonal Antibody (EM92)-eFluor 660 (5 µL/sample at 1:20 dilution)	ThermoFisher Scientific	50-5841-82
27	SOX2 Monoclonal Antibody (Btjce), Alexa Fluor™ 488 (5 µL/sample at 1:20 dilution)	ThermoFisher Scientific	53-9811-82
28	Brilliant Violet 421™ anti-human CD34 Antibody (5 µL/sample at 1:20 dilution)	Biolegend	343610
29	CD270 (HVEM) Monoclonal Antibody (LH1), PE (5 µL/sample at 1:20 dilution)	ThermoFisher Scientific	12-5962-80
30	CD274 (PD-L1, B7-H1) Monoclonal Antibody (MIH1), eFluor™ 450 (5 µL/sample at 1:20 dilution)	ThermoFisher Scientific	48-5983-42
31	APC anti-human IgD Antibody (5 µL/sample at 1:20 dilution)	Biolegend	348222
32	PE anti-human CD27 Antibody (5 µL/sample at 1:20 dilution)	Biolegend	302808
33	IgG1 Mouse anti-human FITC (10 µL/sample at 1:10 dilution)	Beckman Coulter	IM0639U
34	IgG1 Mouse anti-human PE (10 µL/sample at 1:10 dilution)	Beckman Coulter	IM0670U
35	IgG1 Mouse anti-human APC (10 µL/sample at 1:10 dilution)	Beckman Coulter	IM2475U
36	IgG1 Mouse anti-human PC7 (10 µL/sample at 1:10 dilution)	Beckman Coulter	6607099
37	Mouse IgG1 kappa Isotype Control (P3.6.2.8.1), eFluor™ 450 (5 µL/sample at 1:20 dilution)	ThermoFisher Scientific	48-4714-82
38	Rat IgG2 kappa Isotype Control eFluro-660 (5 µL/sample at 1:20 dilution)	ThermoFisher Scientific	50-4321-82
39	BV421 Mouse IgG1, k isotype Control (5 µL/sample at 1:20 dilution)	BD Biosciences	562438
40	Human BD Fc Block (5 µL/sample at 1:20 dilution)	BD Biosciences	564220
41	PerFix-nc (no centrifuge assay kit)	Beckman Coulter	B31168
42	Dulbecco's Phosphate Buffered Saline	VWR	02-0119-0500

Material and Methods:

Western blot

Cells were lysed with the radioimmunoprecipitation assay (RIPA) buffer for Western blotting as previously described[36]. Cell sample of each group had three biological replicates with two technical replicates for each biological replicate (total N = 6 wells) and was combined together for one-time Western Blotting. Protein concentration was determined with a bicinchoninic acid (BCA) protein assay. The proteins were separated with 10% Tris-HCl gel (Bio-Rad, CA, USA) and transferred to the polyvinylidene fluoride membrane. The membrane was normally incubated with 5% non-fat dry milk (ShopRite, Keasbey, NJ, USA) for 30 mins to block the non-specific binding. The proteins were then blotted overnight with anti-human phospho-Akt and anti-human phospho-Erk1/2 mAbs (Cell Signaling, MA, USA) at 1:2000 dilution, followed by the incubation with anti-rabbit or anti-mouse horseradish peroxidase-conjugated secondary Ab (ThermoFisher scientific) at 1: 10000 dilution at room temp for two hours, respectively, and followed by washing with TBST [1]. The membrane was incubated with the chemiluminescent substrate (ThermoFisher Scientifc, CA, USA) and the chemiluminescent signal was detected by using the ChemiDoc Imaging System (Bio-Rad, CA, USA). β-actin served as the internal controls.
